# Increasing incidence of childhood tumours of the central nervous system in Denmark, 1980–1996

**DOI:** 10.1038/sj.bjc.6603278

**Published:** 2006-07-25

**Authors:** O Raaschou-Nielsen, M Sørensen, H Carstensen, T Jensen, T Bernhardtsen, F Gjerris, K Schmiegelow

**Affiliations:** 1 Institute of Cancer Epidemiology, Danish Cancer Society, Strandboulevarden 49, DK-2100 Copenhagen Ø, Denmark; 2 Paediatric Clinic II, Rigshospitalet, Blegdamsvej 9, 2100 Copenhagen Ø, Denmark; 3 University Clinic of Neurosurgery, Neuroscience Centre, Rigshospitalet, Blegdamsvej 9, 2100 Copenhagen Ø, Denmark

**Keywords:** central nervous system, neoplasms, child, incidence, mortality, magnetic resonance imaging

## Abstract

The registered incidence rate of childhood central nervous system (CNS) tumours has increased in several countries. It is uncertain whether these increases are biologically real or owing to improved diagnostic methods. We explored the medical records of 626 CNS tumours diagnosed in Danish children between 1980 and 1996. Population-based registers were used to extract data on mortality and background population. Temporal patterns were analysed by regression techniques. Most tumours were verified by computed tomography (78%) or magnetic resonance imaging (14%). Overall, the incidence rate increased by 2.9% per year (95% confidence interval (CI): 1.3;4.5) and the mortality rate increased by 1.4% per year (95% CI: −0.4;3.3). Among children aged 0–4 years, the survival rate after diagnosis remained almost unchanged, whereas among children aged 5–14 years, the 10-year survival rate improved from 59 to 74%. These data suggest that the incidence rate of CNS tumours among Danish children has truly increased, although alternative explanations cannot be excluded.

Increasing incidence rates of central nervous system (CNS) tumours in children have been reported for several populations in countries where incident cancer cases are registered routinely, that is, in Australia, Italy, Sweden, the United Kingdom and the USA (Mosso *et al*, 1992; McKinney *et al*, 1994; Giles *et al*, 1995; Bunin *et al*, 1996; Gurney *et al*, 1996; Hjalmars *et al*, 1999; McNally *et al*, 2001; Dreifaldt *et al*, 2004). It is uncertain whether these increases are owing to improved diagnostic methods, changes in cancer registration procedures or changes in tumour classification practices, or whether the increases are biologically real and owing to changes in environmental or lifestyle factors (Black, 1998; Smith *et al*, 1998; Forman, 1999; Schechter, 1999; Varner, 1999).

In Sweden, the increase occurred throughout the 20-year period 1973–1992 (Hjalmars *et al*, 1999), covering the introduction of both computed tomography (CT) and magnetic resonance imaging (MRI) as diagnostic methods. In contrast, the increase in the incidence rate of childhood CNS tumours in the United Kingdom occurred mostly before the introduction of MRI (McKinney *et al*, 1994; McNally *et al*, 2001). In the USA, the increase in childhood CNS tumour incidence rate occurred within a few years during the mid-1980s, when MRI became widespread (Steinberg, 1986; Smith *et al*, 1998), and was preceded and followed by relatively stable rates (Gurney *et al*, 1996; Smith *et al*, 1998); at the same time, a slight but steady decrease in specific mortality from CNS tumours in childhood was registered (Linet *et al*, 1999).

So far, the use of MRI for the diagnosis of CNS tumours has been quantified by the number of scanners available (Smith *et al*, 1998) or, in the USA, by the rate of MRI use by Medicare participants (Legler *et al*, 1999). The temporal patterns thus obtained do not, however, necessarily reflect the actual use of MRI in the diagnosis of childhood CNS tumours. In the present study, we explored the temporal patterns of childhood CNS tumour incidence rates in relation to actual use of CT and MRI for diagnosis of the single patient.

## MATERIALS AND METHODS

### Tumour identification

We studied primary CNS tumours (including also nonmalignant types) diagnosed in Denmark during childhood (0–14 years) between 1 January 1980 and 31 December 1996. The year 1980 was chosen to begin the study period before introduction of MRI in Denmark and 1996 was the most recent year with complete data available in the Danish Cancer Registry, when the study was initiated. The tumours had to conform to diagnostic group III of the classification scheme suggested Birch and Marsden (1987), which consists of astrocytoma, medulloblastoma, ependymoma, ‘other glioma’, and ‘miscellaneous intracranial and intraspinal neoplasms’. Central nervous system tumours outside group III of the classification scheme, for example, intracranial germ-cell tumours, were not included. As suggested by the International Agency for Research of Cancer in 1996, the group of medulloblastomas was expanded to include ‘other primitive neuroectodermal tumours’ (Kramarova and Stiller, 1996). Tumours that potentially complied with our inclusion criteria were identified in the population-based Danish Cancer Registry (Storm *et al*, 1997), the population-based Danish Hospital Discharge Registry (Andersen *et al*, 1999) and a clinical database (Gjerris *et al*, 1998), established by a collaboration between the major neurosurgical, pathological, paediatric and radiological hospital departments in Denmark and updated through the 1990s. We used wider criteria than those of Birch and Marsden (1987), when we identified potential CNS tumours from the Danish Hospital Discharge Registry and the clinical database, which minimised the risk for overlooking relevant tumours. We identified 780 potentially relevant tumours and extracted all relevant data by scrutinising their medical records. Eight potential tumours were excluded because no or insufficient medical data could be identified and retrieved.

We examined the medical records for the morphology and topography of the tumour, the date of onset of symptoms, the date of diagnosis and the conclusion drawn from preoperative diagnostic procedures, the date of diagnosis and the presence of hereditary syndromes that are linked to an increased risk for CNS tumours. If the conclusion of the preoperative diagnostic procedures clearly stated that a CNS tumour had been found and this diagnosis was not subsequently disproved, then the tumour was categorised as having been ‘verified’. The first technique used to verify a tumour was defined as *the* verifying diagnostic technique for that tumour. The date of diagnosis was defined as the date of the first diagnostic procedure that raised a suspicion of or verified a tumour. If a tumour was first discovered at autopsy (*n*=4), then the date of death was registered as the date of diagnosis. On the basis of the medical records, each tumour was allocated morphology and topography codes according to the coding system of the International Classification of Diseases for Oncology (ICD-O) (World Health Organisation (WHO), 1976).

Of the 772 potential tumours for which medical records were available, 146 were excluded because the morphology did not qualify as a CNS tumour (e.g. hamartomas and arachnoid cysts) (*n*=100) or because the date of diagnosis was after the 15th birthday of the patient (*n*=46). Thus, 626 children with a CNS tumour were included, of whom 514 had a histologically confirmed specified tumour, 12 had a histologically confirmed but not otherwise specified tumour and 100 had no microscopic confirmation of the tumour, which had been diagnosed only on the basis of diagnostic imaging and clinical symptoms. We received information about the vital status of the 626 children on 31 May 2005 from the Central Population Registry; if they were dead or had emigrated or disappeared, the date of that event was recorded. In addition, we could, up to 31 December 2001, obtain detailed data on the cause of death in the nationwide Danish Mortality Registry (Juel and Helweg-Larsen, 1999), where we identified 278 children who had died during 1980–2001 with a CNS tumour as the underlying cause of death.

### Statistical methods

From the Danish Statistics Bureau, we determined the size of the Danish childhood population by age and gender for each year, and used the data to calculate incidence rates year-by-year and standardised for age in the age groups 0, 1–4, 5–9 and 10–14 years according to the age distribution of the Danish childhood population in 1988, which is the mid-year of the study period. The graphical representation of the results was based on these yearly rates, with a smoothing spline to indicate temporal patterns. The graphical presentation indicated that the incidence rate jumped to a higher level from 1987 to 1988. Therefore, we fitted both a jump model and a linear model to the incidence data. The jump model allowed a constant annual increase (or decrease) from 1980 to 1987, a new level in 1988 and a constant annual increase (or decrease) from 1988 to 1996. We tested if the jump model fitted better to the incidence data than a linear model by use of the likelihood ratio test; both models included variables for sex and four age groups. We further explored the temporal trends in annual incidence and mortality rates by Poisson regression analyses, in which we modelled the effect of calendar time as a linear trend and adjusted for age in the four age intervals. Temporal trends were estimated as the annual change in percentage, with confidence intervals (CI) based on Wald test; likelihood ratio tests were used to test for statistical significance. All statistical tests were two-sided. We used SAS PROC GENMOD (version 8.2) for the estimations.

We used time between onset of symptoms and date of diagnosis as a general indicator of diagnostic efficiency. The temporal trend was analysed by standard linear regression analysis (PROC GLM, SAS version 8.2), with time between onset of symptoms and diagnosis as the dependent variable and year of diagnosis as the independent variable. The distribution of time between onset of symptoms and diagnosis was right-skewed in this material and was therefore transformed by the natural logarithm. In these analyses, we excluded 23 children for whom the date of onset of symptoms was not registered in their medical records. Temporal trends in age at diagnosis were analysed by standard linear regression with age at diagnosis as the dependent variable and year of diagnosis as the independent variable. We investigated the survival of children who received their diagnosis during the first (1980–1987) and last (1988–1996) part of the study period by Kaplan–Meier plots. Differences in survival were tested by a Cox proportional hazard model (PROC PHREG, SAS version 8.2), with adjustment for sex, age (0, 1–4, 5–9 and 10–14 years), morphological type (pilocytic astrocytoma, other astrocytoma, primitive neuroectodermal tumour, ependymoma, other glioma, other) and location in the CNS (cerebellum, cerebrum, brain stem, other). Four CNS tumours first verified at autopsy were included in the survival analyses with an observation time equal to zero.

In a *post hoc* analysis, we used the most recent data from the Danish Cancer Registry to explore childhood CNS tumour incidence rates (age standardised to the 1988 population) for the 5 years (1997–2001) following the study period. Medical records were not available for these patients.

## RESULTS

### CNS tumours in children

Of the 626 incident childhood CNS tumours diagnosed between 1980 and 1996, 29% was located in the cerebellum, 28% in the cerebrum, 13% in the brain stem and 30% in other locations (Table 1); 84% had been histologically verified. Overall, the incidence rate was 39.5 per million population-years, with slightly higher rates among younger children (0–4 years) than among older children (5–14 years). In younger children, the incidence rate was 7% higher among girls than among boys, whereas among older children the incidence rate was 22% higher among boys than among girls. Twenty-five children had Recklinghausen neurofibromatosis and four children had tuberous sclerosis. With regard to tumour type, 39% of the patients had astrocytomas, 18% had primitive neuroectodermal tumours, 9% had ependymomas, 3% had other gliomas and 31% had other tumours, including tumours of unspecified histological type.

### Time trends in incidence rates

**Figure 1:**
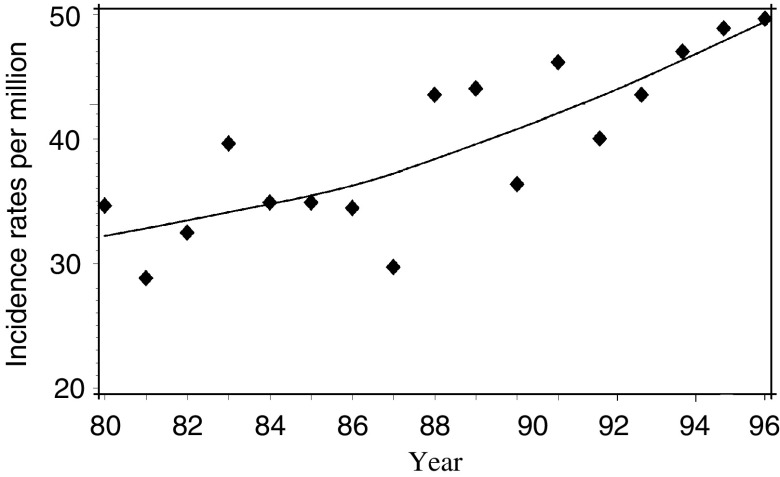
Annual incidence rates (per million) of CNS tumours among Danish children, 1980–1996, age standardised to the Danish childhood population in 1988. A smoothing spline indicates the temporal pattern.

The yearly incidence rate of childhood CNS tumours increased from about 30 to about 50 per million population during the 17-year study period (Figure 1). A jump model did not fit better to the incidence data than a model for a constant linear increase over the whole study period (*P*=0.55). In a linear model, the incidence rate for all CNS tumours increased by 2.9% per year (95% CI: 1.3; 4.5) (Table 2). The incidence rate increased consistently for all morphological subgroups, except for the small subgroup of ‘other gliomas’. The most pronounced increase over the study period was seen for astrocytomas and primitive neuroectodermal tumours. In particular, the incidence of the subgroup of pilocytic astrocytomas increased by almost 12% per year during the study period, whereas those of the other subgroups increased by 1–3% per year. The incidence rates of tumours of all four sites in the CNS increased, but most markedly for tumours of the cerebrum and of ‘other’ locations in the CNS (Table 2).

The incidence rate increased by 3.9% (95% CI: 1.7;6.2 %) per year for boys and by 1.7% (95% CI: −0.6;4.1%) for girls, but the proportion of cases in boys during the first half of the study period did not differ significantly from that in the last half (51% of 264 cases *vs* 56% of 362 cases, *P*=0.25).

The incidence rate increased by 3.7% (95% CI: 0.9;6.5%) per year for children aged 0–4 years and by 2.4% (95% CI: 0.5;4.5 %) per year for children aged 5–14 years. Hence, the proportion of cases in 0–4 year-old children increased from 30% during 1980–1987 to 38% during 1988–1996 (*P*=0.03), and the mean age at diagnosis fell from 7.6 years in the first half of the study period (1980–1987) to 6.9 years in the last half (1988–1996). The annual decrease in the mean age throughout the period was estimated to be 0.11 year (95% CI: −0.20;–0.02 year) per calendar year.

The proportion of CNS tumours in children with a hereditary syndrome was 4% (*n*=10) in the first half of the study period and 5% (*n*=19) in the last half.

A *post hoc* analysis based only on data from the Danish Cancer Registry showed that the age-standardised incidence rates for childhood CNS tumours were 49.4, 42.9, 48.7, 39.8 and 45.4 for the 5 years following the present study (1997–2001).

### Diagnostic procedures

**Figure 2:**
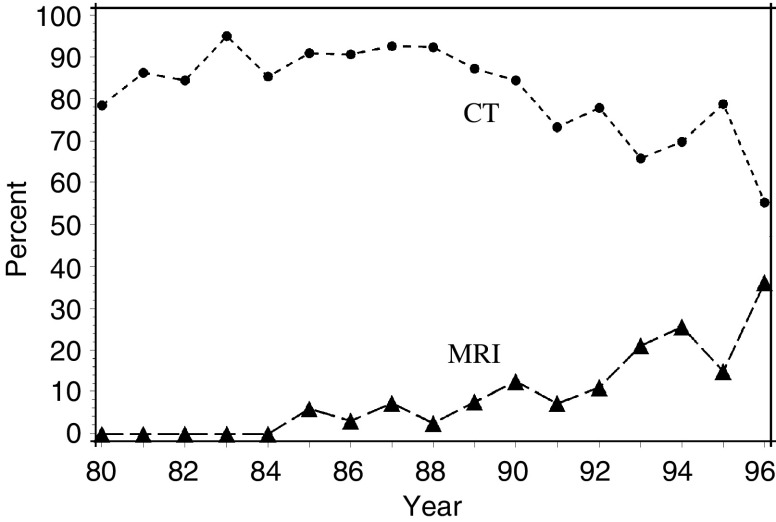
Percentage of childhood CNS tumours diagnosed in Denmark, 1980–1996, for which CT scanning (dots) or MR scanning (triangles), respectively, was the first method used to verify the tumour. Ten tumours were excluded as they had been verified by both CT and MRI on the same day.

According to information provided by Danish hospitals, the first CT scanners were introduced in the largest hospitals during the mid-1970s, and CT scanners gradually became available at the other hospitals during the 1980s and 1990s. The first MR scanner was introduced in Denmark in 1984 and the next four in 1989; the MRI capacity then gradually increased during the 1990s. Examination of the medical records showed that MRI was used to verify a childhood CNS tumour for the first time in Denmark in 1985, and the proportion of CNS tumours verified in this way increased slowly throughout the study period. Computed tomography remained the procedure of choice for verifying the majority of CNS tumours throughout the study period (Figure 2). Tumours classified as ‘others’, including ‘not histologically verified’, represented the largest proportion of tumours verified by MRI, whereas pilocytic astrocytoma and primitive neuroectodermal tumours represented the smallest proportions. Tumours of the brain stem and ‘other location’ were most frequently verified by MRI, whereas tumours of the cerebellum were most rarely verified in this way (Table 3).

The number of days between the onset of symptoms and diagnosis decreased during the study period, with a mean of 360 days in the period 1980–1987 (maximum: 12.7 years) and 257 days in the period 1988–1996 (maximum: 12.8 years), for an estimated 2.7% decrease per year (95% CI: 0.3;5.2%) for the entire study period (Table 4). The annual decrease was 5% for nonpilocytic astrocytomas and ependymomas, whereas there was virtually no change over time for pilocytic astrocytomas. Tumours of the cerebrum were associated with the most pronounced decrease in time between the onset of symptoms and diagnosis.

### CNS tumour-specific mortality rates

The results indicated an increase in the CNS tumour-specific mortality rate for children between 1980 and 2001. In a linear model, the annual increase was 1.4% (95% CI: −0.4;3.3%; *P*=0.12; *n*=278). The increase was slightly higher for children aged 0–4 years, among whom the mortality rate increased by 1.8% per year (95% CI: −1.3;5.0%; *P*=0.26; *n*=99) than for children aged 5–14 years with an estimated annual increase of 1.3% (95% CI: −1.0;3.6% increase; *P*=0.28; *n*=179).

### Survival

**Figure 3:**
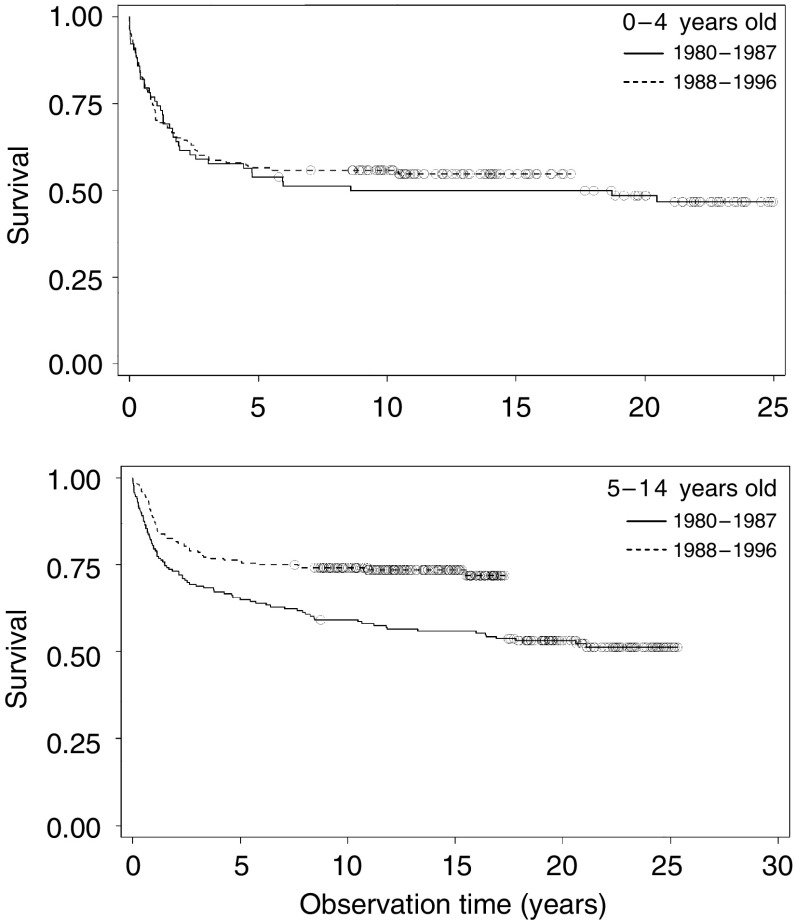
Kaplan–Meier plots of survival after a diagnosis of a CNS tumour in Denmark in 1980–1987 and 1988–1996. The upper panel shows children aged 0–4 years, and the lower panel represents children aged 5–14 years at the time of diagnosis.

Survival after a diagnosis of a CNS tumour was almost constant for children aged 0–4 years throughout the study (*P*=0.99), whereas the survival of children aged 5–14 years improved significantly between the first and the last parts of the study (*P*<0.0001) (Figure 3), such that the 10-year survival increased from 59 to 74%.

## DISCUSSION

This study shows that the incidence rate of CNS tumours among Danish children increased significantly between 1980 and 1996, both for the young (0–4 years) and the older children, with increases for all major morphological and topographical subgroups. Overall, these findings agree with those of most previous studies (Blair and Birch, 1994; McKinney *et al*, 1994; Bunin *et al*, 1996; Gurney *et al*, 1996; Hjalmars *et al*, 1999; McNally *et al*, 2001; Dreifaldt *et al*, 2004), although differences have been found. Thus, a British study showed a stable rate of ependymomas (McNally *et al*, 2001), one study in the USA showed a stable rate of primitive neuroectodermal tumours (Gurney *et al*, 1996) and another study in the USA, spanning 21 years, showed that increases in the incidence rates of various morphological subgroups depends on the period studied (Linet *et al*, 1999). Several aspects of and possible explanations for the increase in incidence rates and methodological aspects of the present study must be considered.

First, the number of CNS tumours analysed in the present study (*n*=626) limited the statistical power for some of the subgroup analyses and precluded analyses of combinations of morphological and topographical subgroups, as was carried out in a larger data set (Smith *et al*, 1998). However, the limited number of tumours facilitated the detailed registration of diagnostic method, time of onset of symptoms and time of diagnosis for each child, which gave us a unique and precise picture of the temporal pattern of use and efficiency of diagnostic methods and the true mortality and survival rates in the present population-based study.

Second, changes in the reliability of tumour registration and disease classification can influence registered incidence rates of CNS tumours. Cases were identified from a nationwide, clinical CNS tumour database and two independent population-based registers, both of which benefit from mandatory registration, resulting in virtually complete case ascertainment. In addition, use of medical records, including reports on pathology and neuroimaging, ensured better validity of the diagnoses than cancer registry data alone (McKinney *et al*, 1994; Thorsteinsson *et al*, 2005). Furthermore, the same persons coded all the tumours according to the ICD-O to ensure consistent coding practice. The registration procedure in the Danish Cancer Registry, which had relied on voluntary notification of incident cancer cases from 1943, became compulsory from 1987 (Storm, 1991). The procedures, including registration of benign CNS tumours, were not changed, but the change could have led to a more complete registration. However, previous analyses of the change from voluntary to compulsory reporting have indicated no overall effect on the number of cancers reported (Storm *et al*, 1997). Furthermore, the Danish Cancer Registry is only one of three sources used to identify the CNS tumours in the present study, and the two other sources would not be affected by the change to compulsory notification to the Danish Cancer Registry. Finally, 1987 was the first year where the Danish Cancer Registry used the Danish Hospital Discharge Registry as an additional source to identify incident cancer cases, which led to a slight increase in the completeness of the Cancer Registry from 1987 (Storm *et al*, 1991). However, in the present study, we also identified potential childhood CNS tumours in the Danish Hospital Discharge Registry for the years 1980 to 1986 to ensure consistent data sources for the present study over the whole study period from 1980 to 1996.

Third, the greater increase in the incidence rate of slowly growing, benign tumours than of more aggressive tumours might indicate that heightened awareness of such tumours, improved diagnostic methods or improved access to diagnosis could explain the increase in childhood CNS tumour incidence, as these slowly growing tumours might otherwise have remained undetected, perhaps throughout life (Gurney *et al*, 1996; Smith *et al*, 1998). Accordingly, one Swedish study (Dreifaldt *et al*, 2004) also found a greater increase in the incidence rate of benign CNS tumours than of other types of CNS tumours, whereas another Swedish study (Hjalmars *et al*, 1999) found that the incidence rate increased to a greater extent for grade 3–4 than for grade 1–2 astrocytomas. We found the most pronounced increase in the annual incidence rate of slowly growing pilocytic astrocytomas, which have a good prognosis (Gjerris *et al*, 1998; Fernandez *et al*, 2003); however, the time between the onset of symptoms and diagnosis of pilocytic astrocytoma remained stable throughout the study, suggesting that earlier detection played only a minor role, if any. Furthermore, the second highest increase in annual incidence rate was for aggressive primitive neuroectodermal tumours, which have a poor prognosis (Lannering *et al*, 1990; Gjerris *et al*, 1998; Alston *et al*, 2003; Viscomi *et al*, 2003), with only a modest decrease in time between the onset of symptoms and diagnosis during the study period. Finally, the incidences of tumours in all the major morphological subgroups increased, as did that of tumours that were not histologically verified, although they continued to constitute a minor proportion of CNS tumours. We cannot rule out the possibility that changes in tumour classification and new histomorphological analyses could have influenced the incidence rates of specific histological subgroups over time, but this would not explain the general pattern of increasing incidence rates in the present study. Moreover, histopathological re-evaluation and homogeneous morphological classification of all CNS tumours in a British series excluded temporal changes in classification practices as a plausible explanation for the observed increase in the incidence rate (McNally *et al*, 2001).

Fourth, improved access to MRI could change the registered incidence even if the biological incidence is unchanged. Thus, the introduction and use of MRI have been suggested as a possible explanation for registered increases in the incidence rate for childhood CNS tumours, supported by a jump in the incidence rate during the few year where MRI was introduced (Black, 1998; Smith *et al*, 1998). This explanation is not, however, supported by the detailed data presented in our study. A jump model did not fit the incidence data better than a linear model and the proportion of CNS tumours verified by MRI remained relatively low throughout the study period. Thus, fluctuations in the annual incidence rates, for example, from 1987 to 1988, could well represent stochastic variation. Furthermore, the morphological tumour types (pilocytic astrocytomas and primitive neuroectodermal tumours) for which the incidence rates increased most markedly were rarely initially verified by MRI. This fact does not exclude the possibility that wider access to MRI and improvements in CT scanners during the 1980s and 1990s might have increased the diagnosis of a few small tumours that would otherwise have remained undetected. The overall increase in both CT and MRI capacity in Denmark during the study period expanded their use for patients with focal epilepsy and nonspecific CNS symptoms, including endocrine disturbances and behavioural changes, although no data are available to quantify this effect.

Fifth, easier access to CT and MRI scans would be expected to shorten the interval between emergence of symptoms and diagnosis and to reduce the age at diagnosis. The incidence would be increased in the youngest age group because age at diagnosis cannot be displaced further downwards for this group. In agreement with this hypothesis, some (Bunin *et al*, 1996; Gurney *et al*, 1996) but not all (Smith *et al*, 1998) previous studies as well as ours indicate a more pronounced increase in the incidence rate among children aged 0–4 years than among those aged 5–14 years. As the incidence also increased in the older age groups, however, this explanation is insufficient for the present results.

Sixth, and most important, if readier access to CT or MRI scans led to a registered but not a biologically based increase in the incidence of childhood CNS tumours, one would expect an improved survival and an unchanged or even reduced specific mortality rate. Most previous studies (Mosso *et al*, 1992; Linet *et al*, 1999; Kaatsch *et al*, 2001; Gonzalez *et al*, 2004), but not all (Shugg *et al*, 1994) have found decreases in mortality from CNS tumours in childhood over the past three decades, probably owing to improved therapy. In the present study, however, we observed an annual 1.4% increase in mortality rate (younger children 1.8%; older children 1.3%), which indeed supports that the observed annual 2.9% increase in incidence rate (younger children: 3.7%; older children: 2.4%) is biologically true. On the other hand, the observed increased survival after a CNS tumour among 5–14-year-old children is compatible with both improved therapy and improved diagnostic methods leading to the detection of tumours that would not previously have been detected before the 15th birthday, but none of these explanations could explain the observed increase in the mortality rate.

In summary, the results of the present study suggest that the incidence rate of CNS tumours among Danish children has truly increased, although additional aspects of the increasing incidence rate must be considered.

## Figures and Tables

**Table 1:** Numbers and crude rates (per million population-years) of incident CNS tumours in Denmark, 1980–1996, by age, gender, tumour morphology and location in the brain

	**Age 0–14 years**						**Age 0–4 years**						**Age 5–14 years**					
	**Both sexes**		**Boys**		**Girls**		**Both sexes**		**Boys**		**Girls**		**Both sexes**		**Boys**		**Girls**	
**Tumour definition**	***n***	**Rate**	***n***	**Rate**	***n***	**Rate**	***n***	**Rate**	***n***	**Rate**	***n***	**Rate**	***n***	**Rate**	***n***	**Rate**	***n***	**Rate**
All CNS tumours	626	39.5	337	41.5	289	37.3	216	42.8	107	41.4	109	44.2	410	37.9	230	41.6	180	34.1
*Tumour morphology*																		
Astrocytomas	247	15.6	123	15.2	124	16.0	83	16.4	39	15.1	44	17.9	164	15.2	84	15.2	80	15.1
Primitive neuroectodermal tumours	111	7.0	64	7.9	47	6.1	42	8.3	19	7.3	23	9.3	69	6.4	45	8.1	24	4.5
Ependymomas	58	3.7	31	3.8	27	3.5	33	6.5	16	6.2	17	6.9	25	2.3	15	2.7	10	1.9
Other gliomas	20	1.3	9	1.1	11	1.4	3	0.6	0	0	3	1.2	17	1.6	9	1.6	8	1.5
Others^a^	190	12.0	110	13.6	80	10.3	55	10.9	33	12.8	22	8.9	135	12.5	77	13.9	58	11.0
																		
*Tumour topography*																		
Cerebellum	183	11.5	100	12.3	83	10.7	64	12.7	30	11.6	34	13.8	119	11.0	70	12.7	49	9.3
Cerebrum	175	11.0	97	12.0	78	10.1	62	12.3	35	13.5	27	11.0	113	10.5	62	11.2	51	9.7
Brain stem	79	5.0	44	5.4	35	4.5	21	4.2	10	3.9	11	4.5	58	5.4	34	6.2	24	4.5
Other locations	189	11.9	96	11.8	93	12.0	69	13.7	32	12.4	37	15.0	120	11.1	64	11.6	56	10.6

CNS, central nervous system.

a Including those without histological confirmation.

**Table 2:** Annual changes in CNS tumour incidence rates in Danish children, 1980–1996

**Tumour definition**	***n***	**Percent change in incidence rate per year (95% CI)**	***P*-value**
All CNS tumours	626	2.9 (1.3;4.5)	0.0005
			
*Tumour morphology*			
Astrocytoma	247	4.4 (1.8;7.0)	0.0009
Pilocytic astrocytomas	66	11.9 (6.2;17.9)	< 0.0001
Other astrocytomas^a^	181	1.9 (– 1.0;4.9)	0.21
Primitive neuroectodermal tumours	111	2.9 (–0.9;6.8)	0.13
Ependymoma	58	1.8 (–3.3;6.9)	0.49
Other glioma	20	–0.7 (–9.2;8.5)	0.87
Others	190	1.6 (–1.3;4.6)	0.27
Not histologically verified	100	1.0 (–2.9;5.1)	0.61
			
*Tumour topography*			
Cerebellum	183	1.3 (–1.6;4.2)	0.40
Cerebrum	175	4.6 (1.5;7.8)	0.003
Brain stem	79	2.1 (–2.3;6.7)	0.35
Other locations	189	3.2 (0.3;6.2)	0.03

CI, confidence interval; CNS, central nervous system.

a Including astrocytomas, not otherwise specified.

**Table 3:** Methods for verification of 454 childhood CNS tumours in Denmark in the period 1985–1996

	**CT**		**MRI**		**Others^a^**	
**Tumour definition**	**%**	***n***	**%**	***n***	**%**	***n***
All CNS tumours	78	355	14	62	8	37
						
*Tumour morphology*						
Astrocytoma	79	147	13	24	8	14
Pilocytic astrocytoma	89	47	6	3	6	3
Other astrocytomas^b^	76	100	16	21	8	11
Primitive neuroectodermal tumours	89	73	5	4	6	5
Ependymoma	77	30	13	5	10	4
Other glioma	57	8	14	2	29	4
Others	72	97	20	27	7	10
Not histologically verified	71	50	26	18	3	2
						
*Tumour topography*						
Cerebellum	89	116	5	6	6	8
Cerebrum	77	103	12	16	11	15
Brain stem	71	40	23	13	5	3
Other locations	72	96	20	27	8	11

CT, computed tomography; CNS, central nervous system; MRI, magnetic resonance imaging.

a Including histological investigation (*n*=11), autopsy (*n*=3), myelography (*n*=3) and ultrasound (*n*=1), more than one method verified the tumour on the same day (*n*=13) and unknown owing to missing results from the first scanning in the medical records (*n*=6).

b Including astrocytomas, not otherwise specified.

**Table 4:** Percentage annual change in time from the onset of symptoms to diagnosis for 603 childhood CNS tumours in Denmark, 1980–1996

**Tumour definition**	***n***	**% change per year**	**95% CI**	***P*-value**
All CNS tumours	603	−2.7	−5.2;−0.3	0.03
				
*Tumour morphology*				
Astrocytoma	241	−3.6	−7.3;0.1	0.06
Pilocytic astrocytoma	64	−0.3	–8.1;8.2	0.95
Other astrocytomas^a^	177	−4.6	−8.7;−0.2	0.04
Primitive neuroectodermal tumours	108	−1.8	−6.2;2.9	0.45
Ependymoma	53	−4.8	−10.5;1.3	0.13
Other glioma	18	−18.6	−31.8;−2.9	0.04
Others	183	1.1	−4.0;6.5	0.69
Not histologically verified	96	−1.7	−8.7;5.7	0.64
				
*Tumour topography*				
Cerebellum	176	−1.9	−5.8;2.1	0.34
Cerebrum	170	−7.1	−11.7;−2.3	0.005
Brain stem	78	−1.8	−8.0;4.8	0.59
Other locations	179	−0.4	−5.0;4.4	0.86

CI, confidence interval; CNS, central nervous system.

a Including astrocytomas, not otherwise specified.
